# KLF15 transcriptionally activates *LINC00689* to inhibit colorectal cancer development

**DOI:** 10.1038/s42003-023-05757-3

**Published:** 2024-01-25

**Authors:** Yan Cao, Jian Li, Gang Zhang, Hao Fang, Yongliang Du, Yan Liang

**Affiliations:** 1 Department of Nuclear Medicine, Xiangya Third Hospital, Central South University, Changsha, 410013 Hunan Province PR China; 2 Department 2 of Gastrointestinal Surgery, Haikou Hospital Affiliated to Xiangya Medical College of Central South University, Haikou People’s Hospital, Haikou, 570208 Hainan Province PR China

**Keywords:** Genetics, Oncology

## Abstract

Colorectal cancer is a grievous health concern, we have proved long non-coding RNA *LINC00689* is considered as a potential diagnosis biomarker for colorectal cancer, and it is necessary to further investigate its upstream and downstream mechanisms. Here, we show that KLF15, a transcription factor, exhibits the reduced expression in colorectal cancer. KLF15 suppresses the proliferative and metastatic capacities of colorectal cancer cells both in vitro and in vivo by transcriptionally activating *LINC00689*. Subsequently, *LINC00689* recruits PTBP1 protein to enhance the stability of *LATS2* mRNA in the cytoplasm. This stabilization causes the suppression of the YAP1/β-catenin pathway and its target downstream genes. Our findings highlight a regulatory network involving KLF15, *LINC00689*, PTBP1, *LATS2*, and the YAP1/β-catenin pathway in colorectal cancer, shedding light on potential therapeutic targets for colorectal cancer therapy.

## Abstract (web summary)

KLF15 transcriptionally activates *LINC00689*, leading to the stabilization of *LATS2* mRNA by recruiting PTBP1, which in turn suppresses colorectal tumorigenesis by inhibiting oncogenes like YAP1 and β-catenin.

## Introduction

Colorectal cancer (CRC) is one of malignant afflictions burdening people and the third most frequently diagnosed cancer worldwide^1^. Therapeutic outcomes for CRC have improved greatly over the past decade with improvement in surgical techniques and systemic therapies, however, patients with CRC may still suffer from poor prognosis and high mortality rate due to proliferation and distant metastasis^2,3^. Therefore, identifying prognostic factors to develop more effective treatment strategies is a crucial task in CRC management.

Long non-coding RNAs (lncRNAs) show multiple valuable functions, serving as decoys, signals, guides, and scaffolds in various tumorigenic processes and other diseases by interacting with RNAs or proteins^4^. In the studies of CRC, *LINC00460* was reported to be upregulated and facilitated cancer cell proliferation and metastasis through directly scaffolding insulin-like growth factor 2 mRNA binding protein 2 (IGF2BP2) and DHX9 to bind with *HMGA1* mRNA^5^. *LncRNA-cCSC1* promoted the proliferative ability of CRC cells by downregulating *miR-124-3p*^6^. In our earlier research, we presented evidence indicating that *LINC00689* was reduced in CRC, and that it acted as a hindrance to CRC cell chemoresistance, proliferation, and metastasis by mediating *miR-31-5p*/large tumor suppressor kinase 2 (*LATS2*) axis^7^. Therefore, *LINC00689* shows potential as a reliable biomarker for predicting the diagnosis and prognosis of CRC patients. Further exploration into the upstream and downstream regulatory mechanisms of *LINC00689* is necessary.

Kruppel-like factors (KLFs) belong to the zinc-finger family of transcription factors that play vital roles in regulating gene expression and controlling various cellular processes^8^. There is growing evidence that links aberrant KLFs activity or expression to development and progression in practically all types of human cancers^9^. KLF15, a member of KLFs family, has suggested to be participated in diverse life activities and tumorigenesis. Dysregulation of KLF15 was indicated to link to the pathogenesis of multiple malignancies, including lung adenocarcinoma^10^, gastric cancer^11^, and breast cancer^12,13^. Zhu et al. demonstrated the anti-tumor effect of KLF15 in breast cancer through restraining tumor cell proliferation and migration, and inducing cell cycle arrest and cell apoptosis^14^. However, the role of KLF15 in CRC has not been clearly studied. UALCAN database revealed that *KLF15* was downregulated in CRC, and more interestingly, KLF15 was predicted by hTFtarget to bind to the promoter region of *LINC00689*. After linking these data, we then speculate that KLF15 is a potential transcription factor of *LINC00689*. However, the regulatory relationship between them has not been reported.

Due to its RNA-binding function, polypyrimidine tract-binding protein 1 (PTBP1) is involved in practically all stages of mRNA regulation to maintain the stability of the mRNA^15^. During the investigation of CRC, PTBP1 was found to have a regulatory function in tumor cell invasion and facilitate the Warburg effect in CRC^16,17^. LATS2 was indicated to show low expression in CRC by several publications^18,19^. StarBase predicts that there are binding sites between PTBP1 and *LINC00689*, as well as between PTBP1 and *LATS2* mRNA. However, the relationship between these three and the biological function of *LINC00689*/PTBP1/*LATS2* axis have not been investigated. On the other hand, LATS2 is a pivotal kinase of the Yes-associated protein (YAP)/Hippo signaling pathway that ensures the spatial and temporal control of YAP activity, while YAP recruits other factors to induce gene transcription^20^. The LATS2/YAP pathway is implicated in proliferation, apoptosis, and other cellular processes^7,21^. In addition, downregulation of LATS2 was reported to activate YAP1 expression in a variety of cancers^22,23^. Also, the activation of the Wnt/β-catenin pathway by YAP1 facilitated the process of intestinal epithelial cell self-renewal, regeneration, and tumorigenesis^24^.

Taken all these data together, our hypothesis suggested that KLF15 transcriptionally regulated *LINC00689*, which in turn bound to PTBP1, thereby enhancing the stability of *LATS2* mRNA and subsequently elevating the protein level of LATS2. This led to the inhibition of the YAP1/β-catenin signaling, ultimately resulting in the suppression of proliferative and metastatic abilities of CRC cells. Understanding the functional roles and mechanisms of KLF15/*LINC00689*/PTBP1/*LATS2* axis contributes to our knowledge of fundamental cellular processes and provides a foundation about potential therapeutic targets for future clinical research of CRC.

## Results

### KLF15 was downregulated in CRC and positively regulated *LINC00689* level through binding to its promoter

**Fig. 1:**
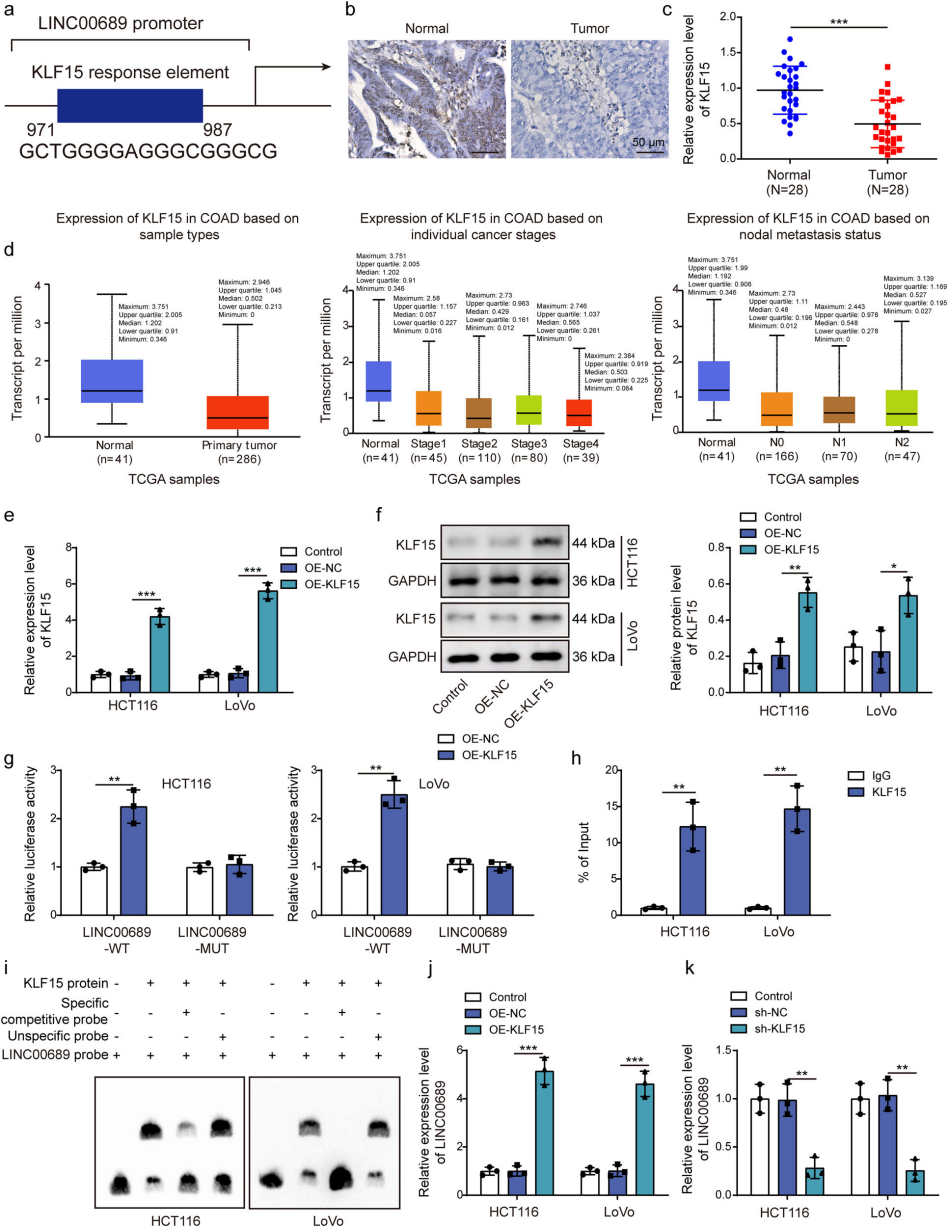
KLF15 was downregulated in CRC and positively regulated *LINC00689* level through binding to its promoter. **a** hTFtarget database predicted the binding sites between transcription factor KLF15 and *LINC00689* promoter. **b**, **c** Immunohistochemistry and RT-qPCR (n = 28) assays measured KLF15 levels in CRC tissues and adjacent non-tumor tissues. Scale bar: 50 μm. **d** UALCAN database indicated *KLF15* relative levels in normal individuals and colon adenocarcinoma (COAD) samples of different clinicopathological features. The variables are sample types, individual cancer stages, and nodal metastasis status. The maximum, upper quartile, median, lower quartile, and minimum values were provided in (**d**). **e**, **f** The transfection efficiency of OE-KLF15 vector was evaluated by RT-qPCR and western blotting in HCT116 and LoVo cells. GAPDH was used as loading control for KLF15 in western blotting. **g** Luciferase reporter, (**h**) CHIP and (**i**) EMSA assays were used to analyze the interaction between KLF15 protein and *LINC00689* promoter. **j**, **k** The relative expression of *LINC00689* was measured by RT-qPCR after transfecting with OE-KLF15 or sh-KLF15 vector. n = 28 biologically independent samples in clinical results. n = 3 biologically independent experiments in in vitro results. Data were presented as mean ± SD except for d, *P < 0.05, **P < 0.01, and ***P < 0.001. KLF15 kruppel-like factor 15, OE overexpression, NC negative control, WT wild type, MUT mutant.

To examine closely upstream and downstream regulatory mechanism of *LINC00689* in CRC pathogenesis, we performed a bioinformatics analysis, which predicted the binding sites between transcription factor KLF15 and *LINC00689* promoter region (Fig. 1a). Next, we observed the downregulation of KLF15 protein (Fig. 1b) and mRNA (Fig. 1c) in patients with CRC. Moreover, *KLF15* relative levels were analyzed in colon adenocarcinoma (COAD) tissues in light of different clinicopathological boundaries from the UALCAN database. *KLF15* was substantially downregulated in COAD tissues based on other variables, such as sample types, individual cancer stages, and nodal metastasis status (Fig. 1d). Subsequently, we transfected plasmids OE-KLF15 into HCT116 and LoVo cells to overexpress KLF15 expression (Fig. 1e, f). The luciferase activity of the *LINC00689*-WT reporter gene was significantly increased after co-transfection with OE-KLF15, while the luciferase activity of the *LINC00689*-MUT reporter gene showed no significant change (Fig. 1g). CHIP experiment further validated the direct binding of KLF15 to *LINC00689* promoter in HCT116 and LoVo cells **(**Fig. 1h). Consistently, EMSA assay indicated the capability of KLF15 protein binding to promoter region of *LINC00689* (Fig. 1i). Furthermore, our findings indicated that overexpression of KLF15 increased *LINC00689* level, while knockdown of KLF15 inhibited its expression **(**Fig. 1j, k). In conclusion, KLF15 enhanced *LINC00689* level in CRC cells through binding to its promoter.

### KLF15 inhibited the proliferative, migratory, and invasive abilities of CRC cells by suppressing YAP1/β-catenin signaling pathway

**Fig. 2:**
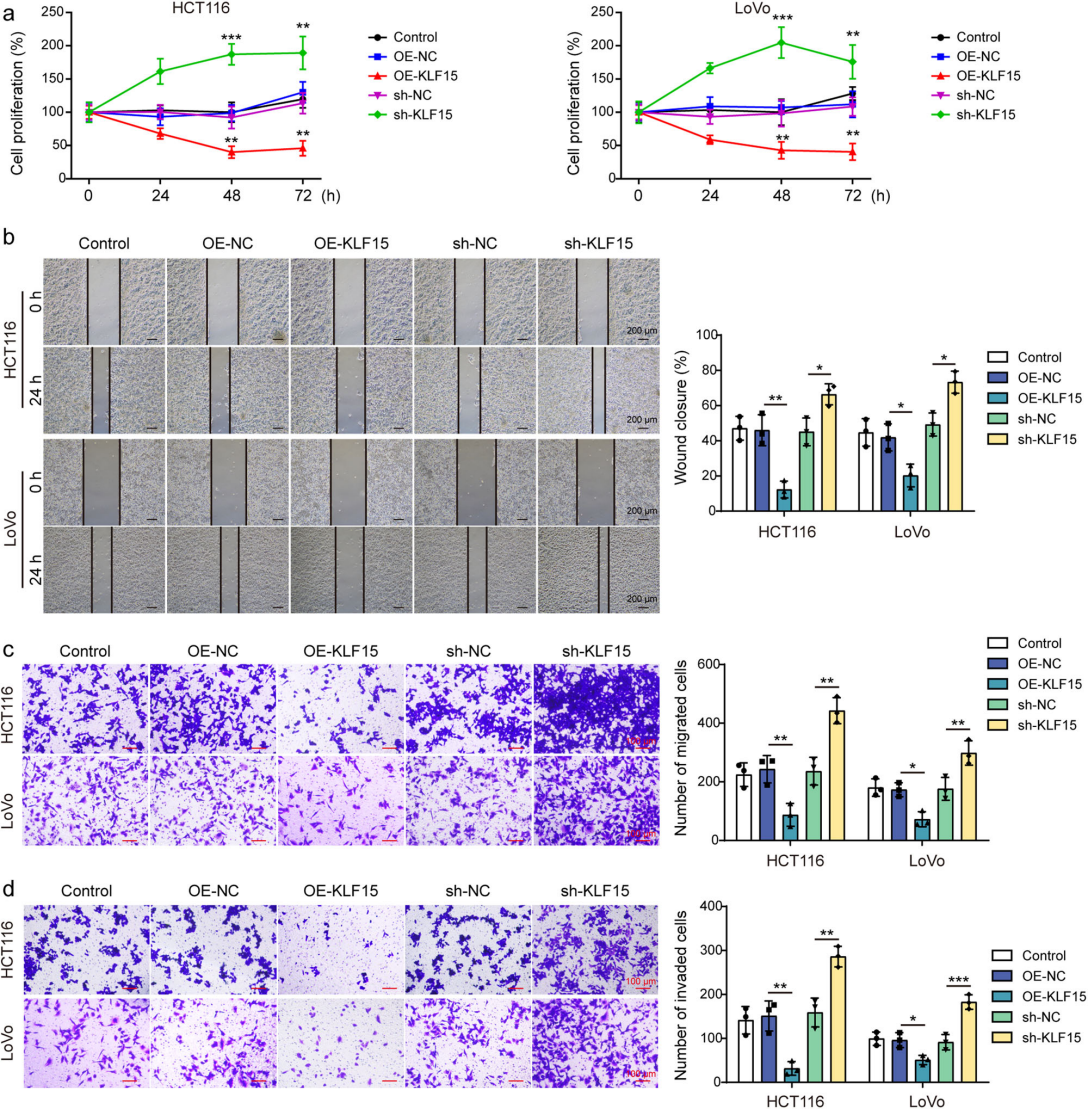
KLF15 inhibited the proliferative, migratory, and invasive abilities of CRC cells. HCT116 and LoVo cells were transfected with OE-KLF15 or sh-KLF15 vector, (**a**) proliferation, (**b**, **c**) migration, and (**d**) invasion abilities of treated cells were assessed by CCK-8, wound healing, and transwell assays, respectively. Scale bar: 200 μm in (**b**) and 100 μm in (**c**/**d**). n = 3 biologically independent experiments in in vitro results. Data were presented as mean ± SD, *P < 0.05, **P < 0.01, and ***P < 0.001. KLF15 kruppel-like factor 15, OE overexpression, NC negative control.

**Fig. 3:**
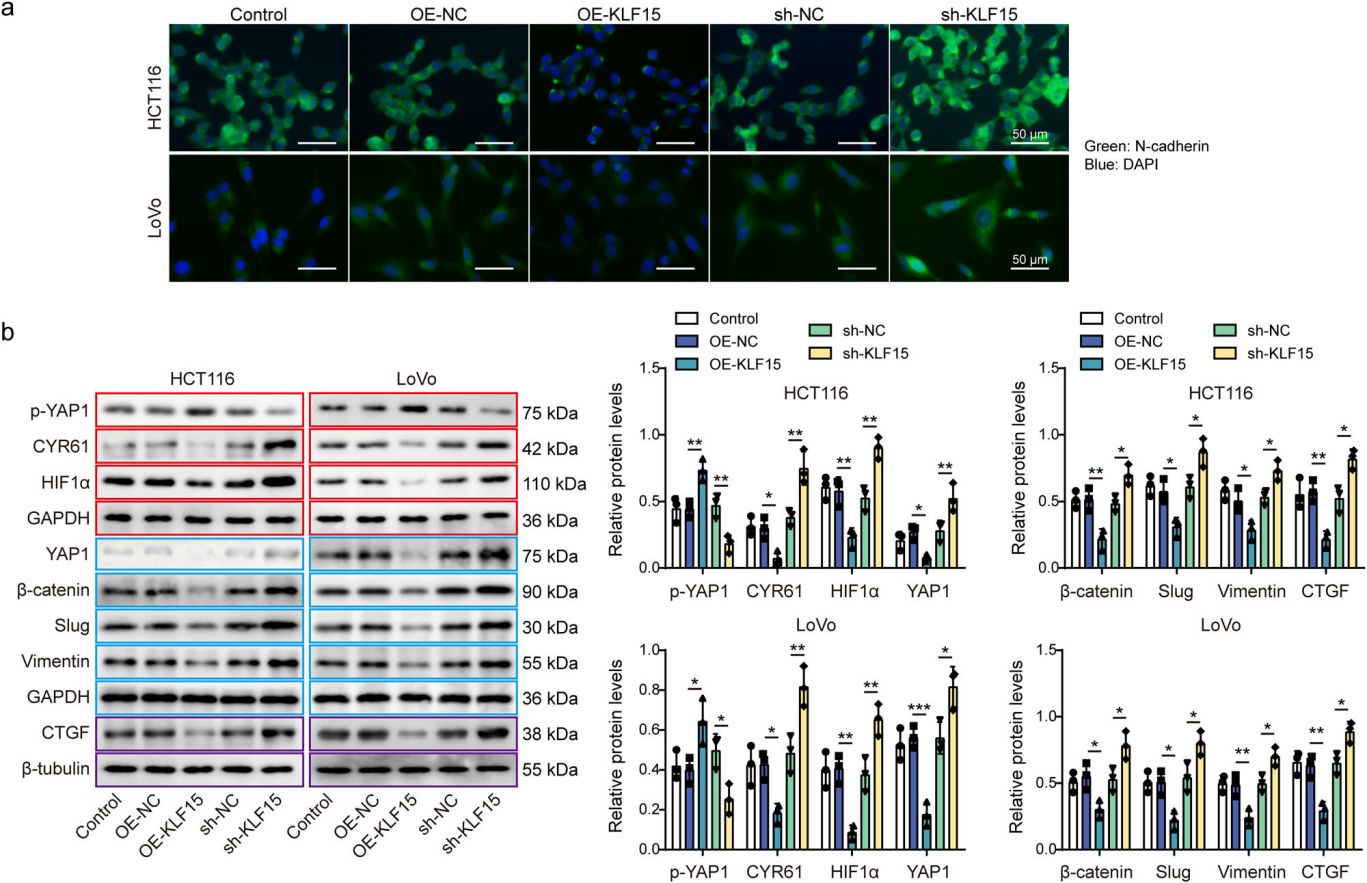
KLF15 inhibited the expression of N-cadherin and YAP1/β-catenin signaling pathway in CRC cells. **a** Immunofluorescence detection of N-cadherin expression in treated cells. Green, N-cadherin; Blue, DAPI. Scale bar: 50 μm. **b** p-YAP1 (S127), YAP1, β-catenin, slug, vimentin, CTGF, CYR61, and HIF1α levels in treated cells were measured by western blotting. β-tubulin was used as loading control for CTGF, and GAPDH was used as loading control for other proteins. Red, blue, or purple outlines denote bands that were derived from the same blot. n = 3 biologically independent experiments in in vitro results. Data were presented as mean ± SD, *P < 0.05, **P < 0.01, and ***P < 0.001. KLF15 kruppel-like factor 15, OE overexpression, NC negative control, YAP1 yes-associated protein 1, CTGF connective tissue growth factor, CYR61 cysteine-rich angiogenic inducer 61, HIF1α hypoxia inducible factor 1α.

Next, we began to determine the biological functions of KLF15 in HCT116 and LoVo cells through gain- and loss-of-function experiments. The results showed that overexpression of KLF15 decreased the proliferative rate of CRC cells (Fig. 2a) and also inhibited the migratory (Fig. 2b, c) and invasive (Fig. 2d) abilities of cancer cells. However, knockdown of KLF15 had the opposite effects on HCT116 and LoVo cells (Fig. 2a–d). N-cadherin has been considered a path-finding molecule involved in invasion, migration, and neurite outgrowth^25^. Here, we found that overexpression of KLF15 reduced the expression of N-cadherin, while knockdown of KLF15 increased its level (Fig. 3a). YAP1/β-catenin pathway has been reported to participate in CRC progression^26^. High levels of connective tissue growth factor (CTGF) and cysteine-rich angiogenic inducer 61 (CYR61) were found in CRC compared with normal colon^27^. Interestingly, YAP1/β-catenin pathway was suggested to increase their expression levels in CRC^28^. Meanwhile, previous publications suggested that YAP1/β-catenin pathway could regulate hypoxia inducible factor 1α (HIF1α) level^29,30^. We, therefore, measured the expression of these proteins to verify the involvement of YAP1/β-catenin pathway. Here, KLF15 overexpression downregulated CTGF, CYR61, HIF1α, YAP1, β-catenin, and epithelial-mesenchymal transition (EMT)-related markers including slug and vimentin, while increasing p-YAP1 (S127) level (Fig. 3b). However, KLF15 knockdown led to opposite results **(**Fig. 3b), suggesting that overexpression of KLF15 suppressed the activation of YAP1/β-catenin pathway and EMT. Taken together, KLF15 was instrumental in regulating CRC cell proliferation and metastasis, and in triggering the activation of YAP1/β-catenin pathway.

### Overexpression of KLF15 inhibited tumor growth in nude mice

**Fig. 4:**
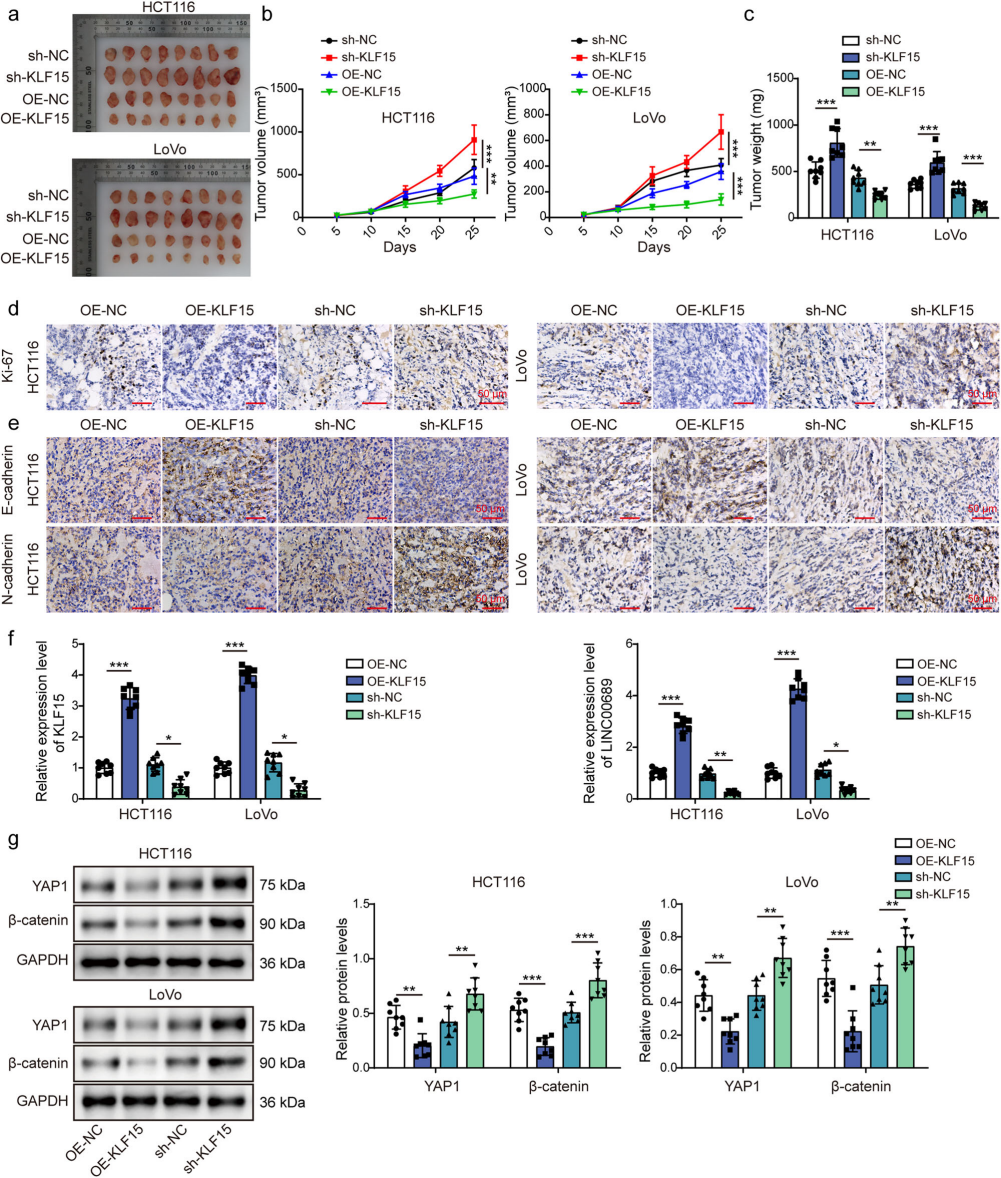
Overexpression of KLF15 inhibited tumor growth in nude mice. HCT116 and LoVo cells overexpressed or silenced KLF15 were injected into xenograft nude mice. After 25 days, mice were executed and tumor tissues were collected for subsequent experiments. **a** Images of tumors in different groups. **b**, **c** Quantification of tumor volume and weight in different groups. **d**, **e** The expression levels of Ki-67, E-cadherin, and N-cadherin in tumor tissues were measured by immunohistochemistry. Scale bar: 50 μm. **f** RT-qPCR evaluated *KLF15* and *LINC00689* levels in tumor tissues. **g** Western blotting determined YAP1 and β-catenin levels in tumor tissues. GAPDH was used as loading control in western blotting. n = 8 biologically independent animals. Data were presented mean ± SD, *P < 0.05, **P < 0.01, and ***P < 0.001. KLF15 kruppel-like factor 15, OE overexpression, NC negative control, YAP1 yes-associated protein 1.

Having confirmed the tumor-inhibiting effects of KLF15 in vitro, we proceeded to validate these effects in vivo. The xenograft tumor model was established to explore tumor growth after subcutaneous injection with HCT116 and LoVo cells stably transfected with OE-KLF15 or sh-KLF15 lentiviral vector. Tumor average volume and weight were repressed after overexpressing KLF15, while the volume and weight in the sh-KLF15 group were increased (Fig. 4a–c). Ki-67 is highly overexpressed in cancer cells and has been proposed as a prognostic marker of cancers^31^. We observed that KLF15 overexpression induced a downregulation of Ki-67 in tumor tissues, while KLF15 knockdown promoted its level **(**Fig. 4d). Moreover, we measured the levels of EMT-related markers, E-cadherin and N-cadherin. Overexpression of KLF15 inhibited N-cadherin level, while promoting E-cadherin level. As expected, KLF15 knockdown obtained opposite results (Fig. 4e). We then observed that injection of CRC cells overexpressed KLF15 increased the expression of KLF15 and *LINC00689* in tumor tissues (Fig. 4f), while decreased YAP1 and β-catenin levels (Fig. 4g). The opposite effects were observed following silencing KLF15 (Fig. 4f, g). Collectively, KLF15 also acted as a tumor suppressor in vivo.

### *LINC00689* knockout increased the proliferative, migratory, and invasive abilities of CRC cells by activating YAP1/β-catenin signaling pathway

**Fig. 5:**
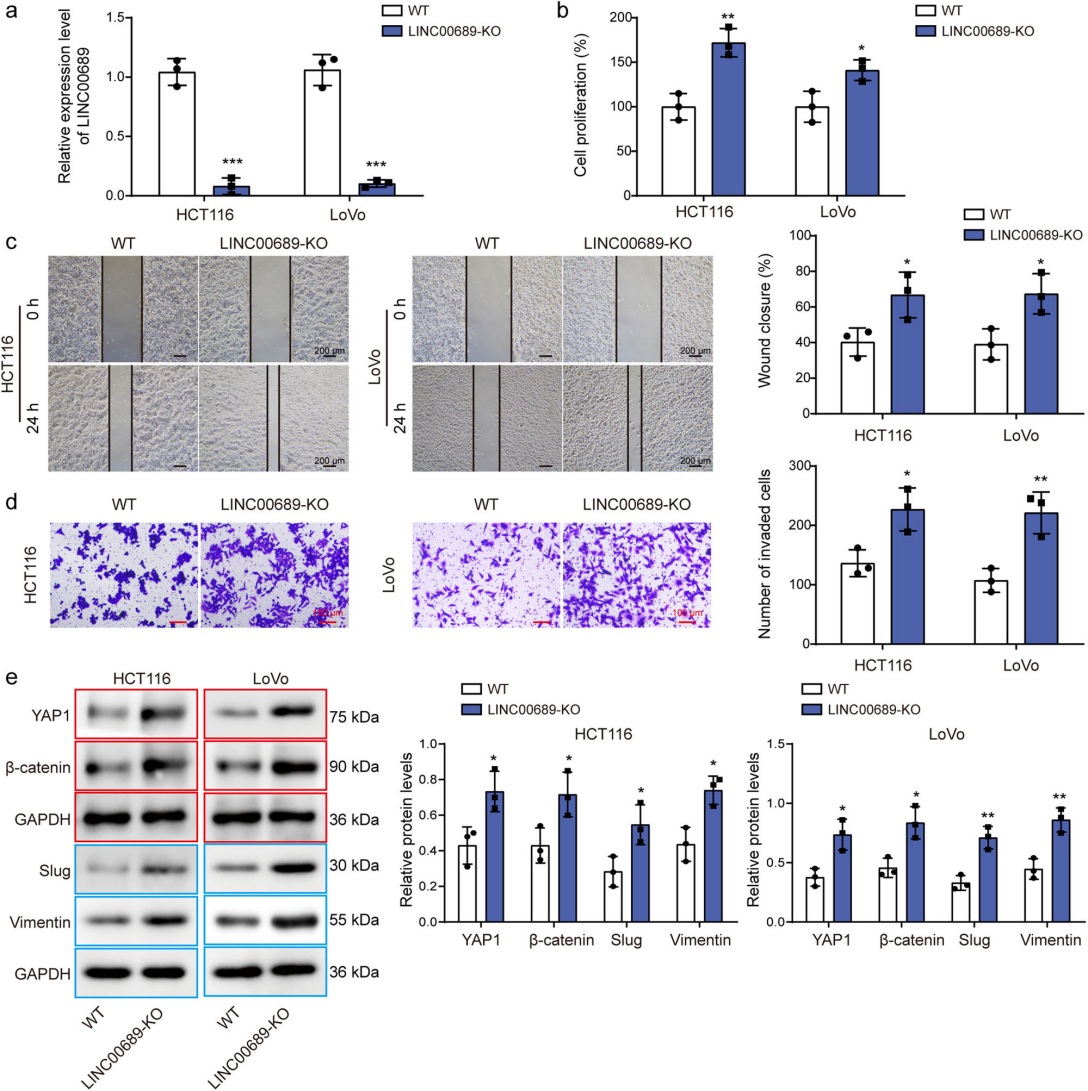
*LINC00689* knockout promoted the proliferative, migratory, and invasive abilities of CRC cells. **a** Relative expression level of *LINC00689* in wild type (WT) and *LINC00689*-KO CRC cells. The (**b**) proliferation, (**c**) migration, and (**d**) invasion abilities of WT and *LINC00689*-KO CRC cells were assessed by CCK-8, wound healing, and transwell assays, respectively. Scale bar: 200 μm in (**c**) and 100 μm in (**d**). **e** YAP1, β-catenin, slug, and vimentin levels in WT and *LINC00689*-KO CRC cells were measured by western blotting. GAPDH was used as loading control in western blotting. Red or blue outlines denote bands that were derived from the same blot. n = 3 biologically independent experiments in in vitro results. Data were presented as mean ± SD, *P < 0.05, **P < 0.01, and ***P < 0.001. YAP1 yes-associated protein 1, WT wild type, KO knockout.

CRISPR/Cas9 technology was utilized to knockout the *LINC00689* gene in HCT116 and LoVo cells. As shown in Fig. 5a, *LINC00689* expression was obviously decreased in *LINC00689*-KO cells. Moreover, knockout of *LINC00689* significantly enhanced the proliferative (Fig. 5b), migratory (Fig. 5c), and invasive (Fig. 5d) capacities of CRC cells. The levels of YAP1, β-catenin, slug, and vimentin in *LINC00689*-KO cells were upregulated (Fig. 5e). These findings suggested that *LINC00689* knockout promoted the growth and metastasis of CRC cell, which might achieve by activating YAP1/β-catenin pathway.

### Knockdown of *LINC00689* abolished the function of KLF15 on CRC cell proliferation and metastasis, and activated YAP1/β-catenin pathway

**Fig. 6:**
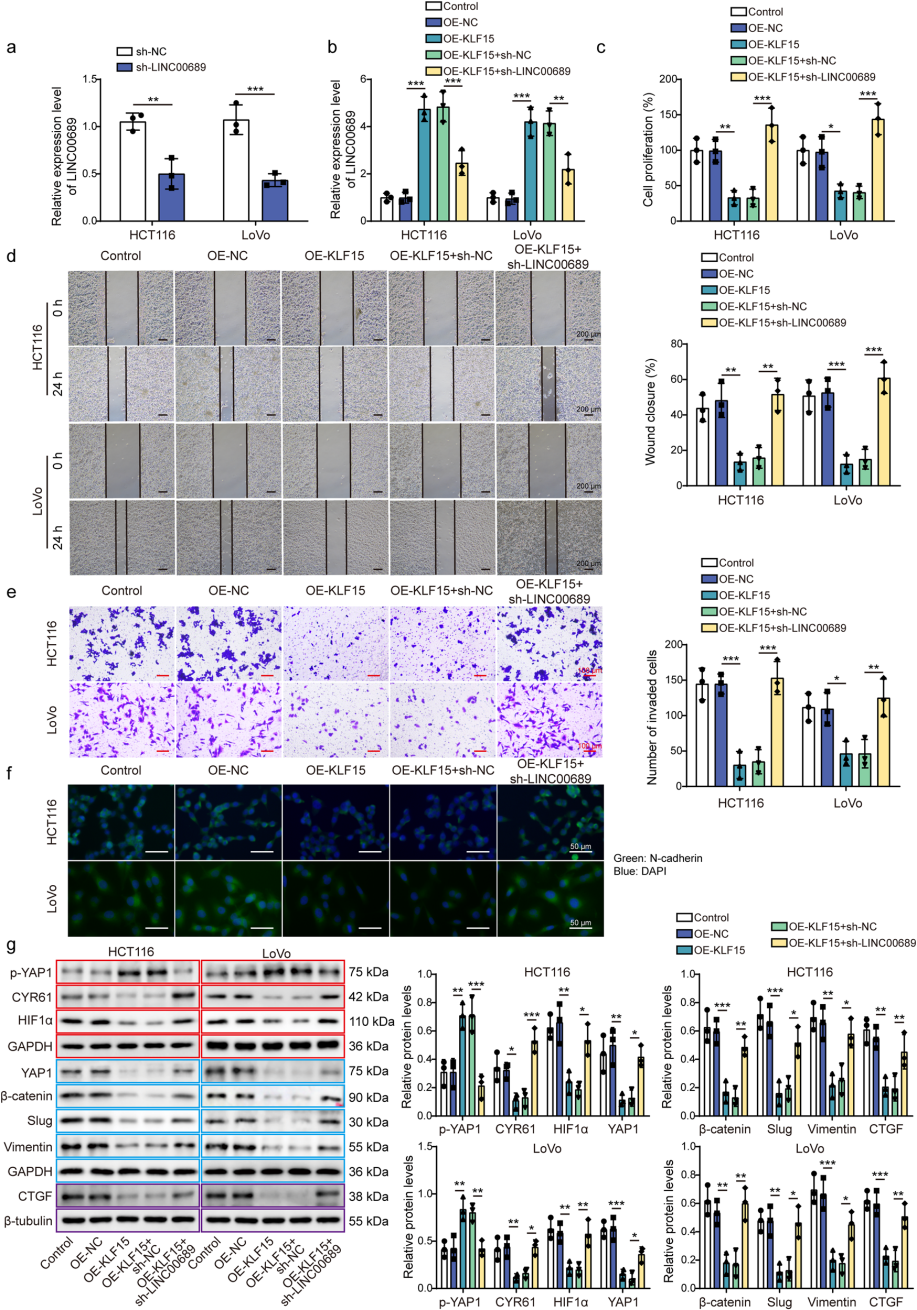
Knockdown of *LINC00689* abolished the function of KLF15 on CRC cell proliferation and metastasis, and activated YAP1/β-catenin pathway. HCT116 and LoVo cells were co-transfected with sh-*LINC00689* and OE-KLF15 vector. Then, **a**, **b** *LINC00689* levels were detected by RT-qPCR. The (**c**) proliferation, (**d**) migration, and (**e**) invasion of treated cells were analyzed by CCK-8, wound healing, and transwell assays, respectively. Scale bar: 200 μm in (**d**) and 100 μm in (**e**). **f** The expression of N-cadherin was evaluated by immunofluorescence. Green, N-cadherin; Blue, DAPI. Scale bar: 50 μm. **g** Western blotting assessed p-YAP1 (S127), YAP1, β-catenin, slug, vimentin, CTGF, CYR61, and HIF1α levels. β-tubulin was used as loading control for CTGF, and GAPDH was used as loading control for other proteins. Red, blue, or purple outlines denote bands that were derived from the same blot. n = 3 biologically independent experiments in in vitro results. Data were presented as mean ± SD, *P < 0.05, **P < 0.01, and ***P < 0.001. KLF15 kruppel-like factor 15, OE overexpression, NC negative control, YAP1 yes-associated protein 1, CTGF connective tissue growth factor, CYR61 cysteine-rich angiogenic inducer 61, HIF1α hypoxia-inducible factor 1α.

Considering the regulatory relationship between KLF15 and *LINC00689*, we next designed and performed rescue experiments to verify the biological functions of KLF15/*LINC00689* axis in CRC. The silenced effect achieved by sh-*LINC00689* was verified in Fig. 6a, and showed the expression of *LINC00689* was decreased. The sh-*LINC00689* plasmid was then transfected into HCT116 and LoVo cells overexpressed KLF15, then we found that the upregulation of *LINC00689* caused by KLF15 overexpression was reduced by silencing *LINC00689* (Fig. 6b). Moreover, knockdown of *LINC00689* reversed the inhibiting effects of KLF15 overexpression on cancer cell proliferation (Fig. 6c), migration (Fig. 6d), and invasion (Fig. 6e). The decreased N-cadherin expression induced by KLF15 overexpression was also upregulated after transfecting with sh-*LINC00689* (Fig. 6f). As expected, knockdown of *LINC00689* abolished the inhibitory effects of KLF15 overexpression on EMT-related markers, the activation of YAP1/β-catenin pathway and its target genes including CTGF, CYR61 and HIF1α (Fig. 6g). Taken together, the KLF15/*LINC00689* axis inhibited malignant phenotypes of CRC cells through suppressing YAP1/β-catenin pathway.

### *LINC00689* recruited PTBP1 protein in CRC cells

**Fig. 7:**
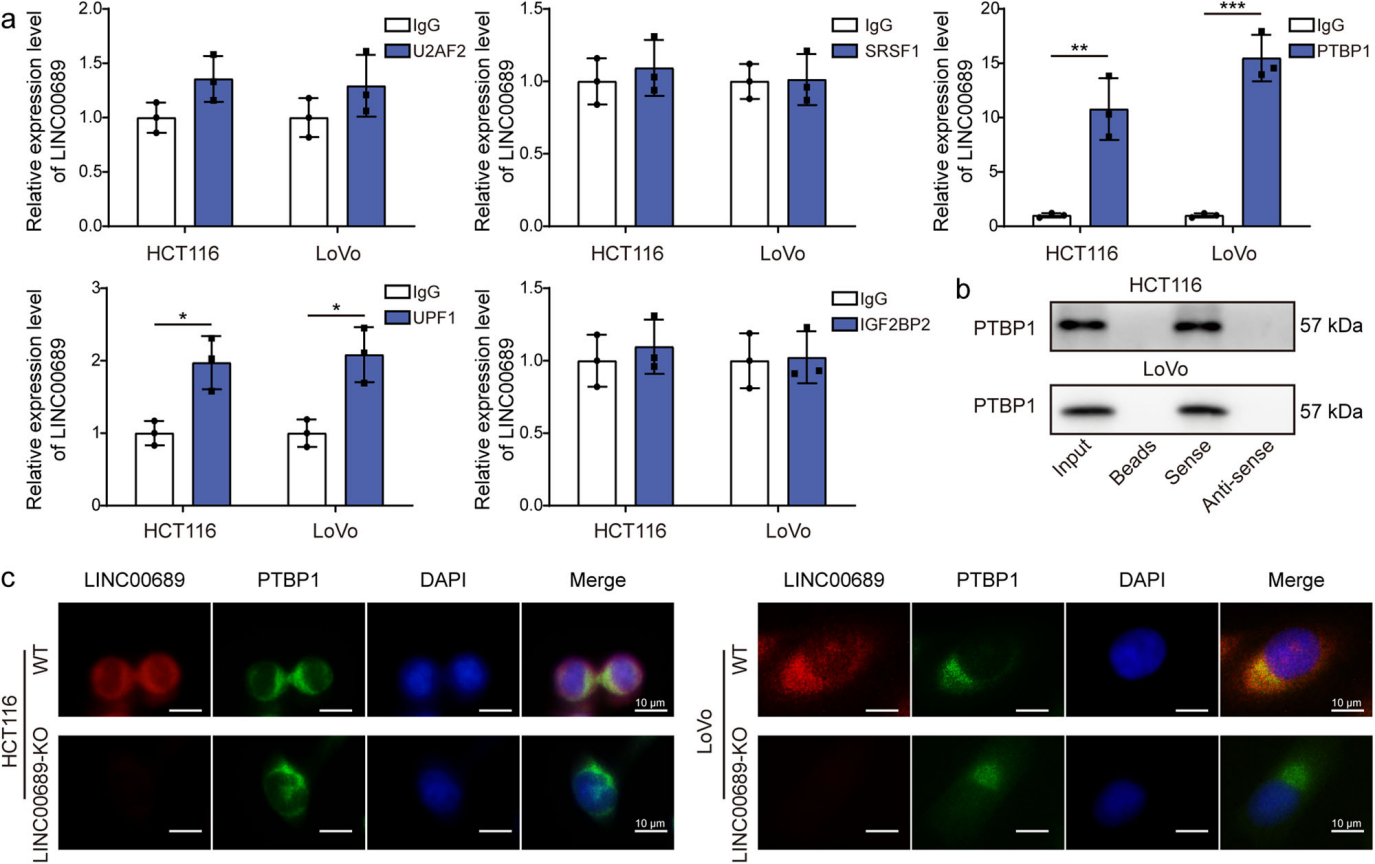
*LINC00689* recruited PTBP1 protein in CRC cells. **a** RIP assay was conducted to confirm the binding of *LINC00689* with five potential proteins (U2AF2, SRSF1, PTBP1, UPF1, and IGF2BP2) predicted by StarBase bioinformatic analysis. **b** The specific association of *LINC00689* and PTBP1 protein was further evaluated using RNA pull-down assay. **c** The co-localization of *LINC00689* and PTBP1 protein in the WT and *LINC00689*-KO HCT116 and LoVo cells was tested using FISH combined immunofluorescence staining. Scale bar: 10 μm. n = 3 biologically independent experiments in in vitro results. Data were presented as mean ± SD, *P < 0.05, **P < 0.01, and ***P < 0.001. U2AF2 U2 small nuclear RNA auxiliary factor 2, SRSF1 serine/arginine-rich splicing factor 1, PTBP1 polypyrimidine tract-binding protein 1, UPF1 upstream frameshift 1, IGF2BP2 insulin-like growth factor 2 mRNA binding protein 2.

Since lncRNAs can implement their functions through binding to proteins^32^, we then began to find the target proteins of *LINC00689*. There were five potential target proteins predicted by StarBase. Through RIP assay, we observed that compared with other co-precipitation groups (U2AF2, SRSF1, UPF1, and IGF2BP2), the enrichment of *LINC00689* in the PTBP1 co-precipitation group showed the most significant increase (Fig. 7a). The data from RNA pull-down assay indicated that *LINC00689* significantly pulled down PTBP1 protein (Fig. 7b). Next, experiments combining FISH and immunofluorescence were conducted to analyze the co-localization of *LINC00689* and PTBP1 in wild type (WT) cells to support their interaction. The results suggested that *LINC00689* was strongly co-localized with PTBP1 protein, particularly in the cytoplasm (Fig. 7c). Overall, *LINC00689* recruited and bound to PTBP1 protein in CRC cells.

### *LINC00689* recruited PTBP1 protein to regulate the stability of *LATS2* mRNA in CRC cells

**Fig. 8:**
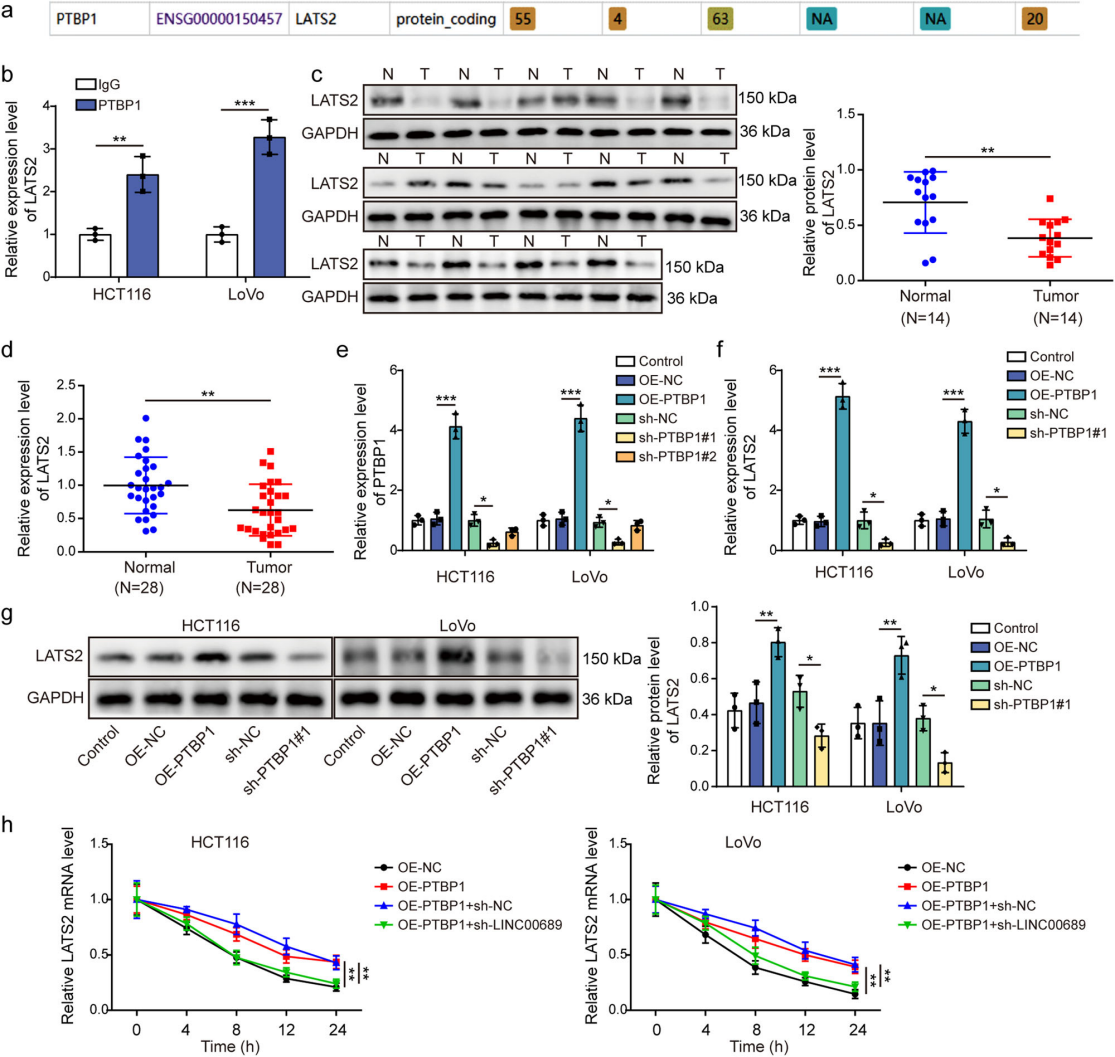
*LINC00689* recruited PTBP1 protein to regulate the stability of *LATS2* mRNA in CRC cells. **a** StarBase bioinformatic tool analyzed potential binding sites between PTBP1 and *LATS2* mRNA. **b** The interaction between PTBP1 and *LATS2* mRNA in HCT116 and LoVo cells was verified by RIP assay. **c**, **d** The protein (n = 14) and mRNA (n = 28) levels of LATS2 in CRC tissues and adjacent normal tissues were determined by western blotting and RT-qPCR. GAPDH was used as loading control in western blotting. **e** The relative expression of *PTBP1* was detected in HCT116 and LoVo cells transfected with OE-PTBP1, sh-PTBP1#1, or sh-PTBP1#2 plasmid by RT-qPCR. **f**, **g** The mRNA and protein expression levels of LATS2 were measured by RT-qPCR and western blotting after overexpressing or knocking down PTBP1. GAPDH was used as loading control in western blotting. **h** *LATS2* mRNA levels in HCT116 and LoVo cells co-transfected with OE-PTBP1+sh-*LINC00689* were examined by RT-qPCR after treatment with 2 μg/mL act-D for 0, 4, 8, 12, and 24 h. n = 28 biologically independent samples in clinical results. n = 3 biologically independent experiments in in vitro results. Data were presented as mean ± SD, *P < 0.05, **P < 0.01, and ***P < 0.001. PTBP1 polypyrimidine tract-binding protein 1, LATS2 large tumor suppressor kinase 2, OE overexpression, NC negative control.

LATS2, a core kinase of the YAP/Hippo pathway, has emerged as a key regulator of several oncogenic or tumor-suppressive regulators, as well as a mediator of EMT in the process of cancer metastasis^33^. Here, the data from StarBase has predicted the potential interaction between PTBP1 and *LATS2* mRNA (Fig. 8a). *LATS2* mRNA was significantly enriched by PTBP1 antibody (Fig. 8b), confirming the target relationship between them. In addition, LATS2 showed downregulation in patients with CRC (Fig. 8c, d). LATS2 mRNA and protein levels were increased in HCT116 and LoVo cells after overexpressing PTBP1, while knockdown of PTBP1 inhibited LATS2 levels (Fig. 8e–g). After overexpressed PTBP1 and silenced *LINC00689*, total RNAs were isolated from HCT116 and LoVo cells after treatment with the Act-D, followed by performing RT-qPCR to determine *LATS2* levels. The half-life of *LATS2* mRNA was enhanced by overexpressing PTBP1, while knockdown of *LINC00689* abolished this stabilizing effect (Fig. 8h). These results promoted us to conclude that *LINC00689* recruited PTBP1 protein to regulate the stability of *LATS2* mRNA to inhibit malignant phenotypes of CRC cells.

### *LINC00689* inhibited proliferation, migration, and invasion of CRC cells through upregulating *LATS2*

**Fig. 9:**
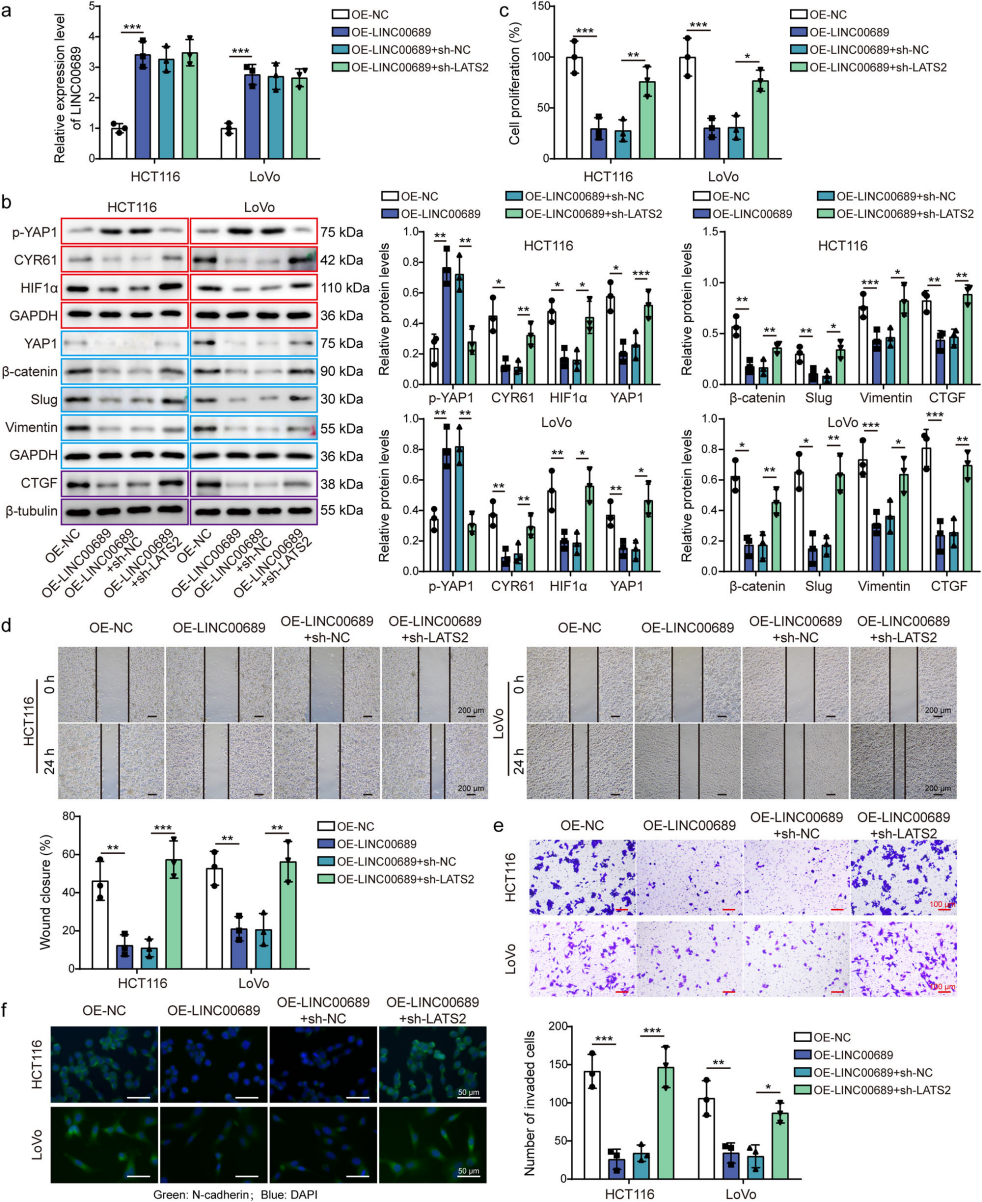
*LINC00689* inhibited proliferation, migration, and invasion of CRC cells through upregulating *LATS2*. **a** *LINC00689*, and (**b**) p-YAP1 (S127), YAP1, β-catenin, slug, vimentin, CTGF, CYR61, and HIF1α protein expression in CRC cells co-transfected with OE-*LINC00689* and sh-LATS2 were examined by RT-qPCR and western blotting, respectively. β-tubulin was used as loading control for CTGF, and GAPDH was used as loading control for other proteins in western blotting. Red, blue, or purple outlines denote bands that were derived from the same blot. **c** CRC cell proliferative, (**d**) migratory, and (**e**) invasive abilities were evaluated after overexpressing *LINC00689* and silencing LATS2 via CCK-8, wound healing, and transwell assays, respectively. Scale bar: 200 μm in d and 100 μm in e. **f** Immunofluorescence detection of N-cadherin expression after simultaneous overexpression of *LINC00689* and knockdown of LATS2. Green, N-cadherin; Blue, DAPI. Scale bar: 50 μm. n = 3 biologically independent experiments in in vitro results. Data were presented as mean ± SD, *P < 0.05, **P < 0.01, and ***P < 0.001. LATS2 large tumor suppressor kinase 2, OE overexpression, NC negative control, YAP1 yes-associated protein 1, CTGF connective tissue growth factor, CYR61 cysteine-rich angiogenic inducer 61, HIF1α hypoxia inducible factor 1α.

Finally, the sh-LATS2 plasmid was transfected into CRC cells overexpressed *LINC00689* to verify the biological function of *LINC00689*/*LATS2* axis. We firstly observed that LATS2 knockdown had no effect on *LINC00689* expression (Fig. 9a). Then, overexpression of *LINC00689* was found to inhibit EMT-related markers (slug and vimentin) and YAP1/β-catenin pathway including upregulating p-YAP1 (S127) and downregulating YAP1, β-catenin, CTGF, CYR61 and HIF1α. Knockdown of LATS2 could reverse these effects (Fig. 9b). Furthermore, overexpression of *LINC00689* repressed the proliferative (Fig. 9c), migratory (Fig. 9d), and invasive (Fig. 9e) capacities of tumor cells, and N-cadherin level (Fig. 9f) in vitro. However, these inhibitory effects were blocked by silencing LATS2 (Fig. 9c–f). In conclusion, *LINC00689* inhibited CRC cell progression by upregulating *LATS2*.

## Discussion

**Fig. 10:**
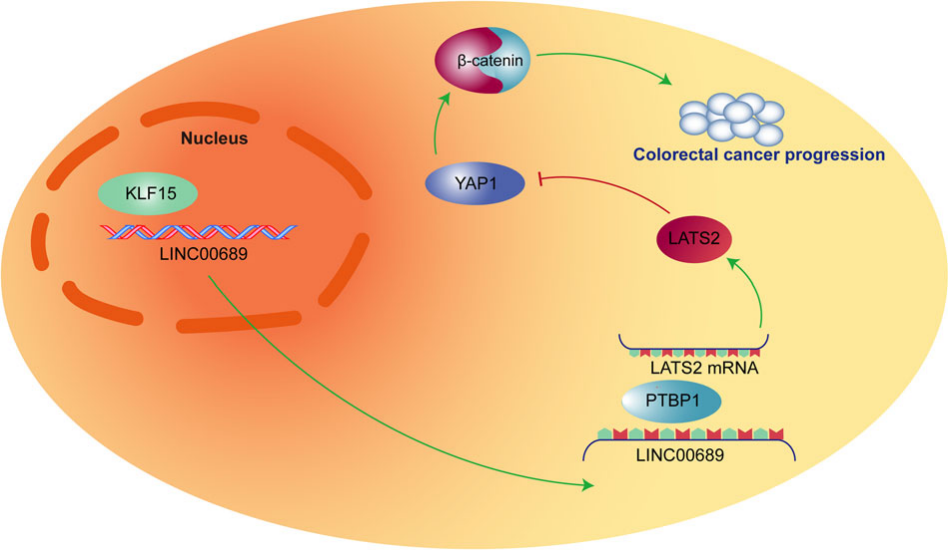
Graphical abstract. Transcription factor KLF15 was found to upregulate *LINC00689* via binding to its promoter, then the upregulated *LINC00689* could enhance the stability of *LATS2* mRNA through interacting with PTBP1 protein, thereby inhibiting CRC development by repressing the activation of YAP1/β-catenin pathway.

The clinical death of CRC individuals has decreased due to the improvement in screening efforts^34^. On the other hand, new therapies have been developed, such as radiotherapy, and neoadjuvant and palliative chemotherapy, which increase the success rate of anti-cancer treatments^35,36^. However, almost half of CRC patients will experience relapse, and the cases that relapse will result in increased mortality due to high metastasis^37^. Thus, developing targeted anti-cancer therapies that disrupt dysregulated signaling in CRC development can enhance patient prognosis. Our previous study revealed that overexpressing *LINC00689* or inhibiting *miR-31-5p* suppressed CRC cell proliferation, chemoresistance, and metastasis by upregulating *LATS2* and inhibiting the YAP1/β-catenin pathway, indicating that *LINC00689* acts as a *miR-31-5p* sponge to inhibit CRC progression^7^. Based on these findings, we further explored *LINC00689*-related upstream and downstream mechanisms in CRC pathogenesis. The results from the present study indicated that transcription factor KLF15 was found to upregulate *LINC00689* via binding to its promoter, then the upregulated *LINC00689* could enhance the stability of *LATS2* mRNA through interacting with PTBP1 protein, thereby inhibiting CRC development by repressing the activation of YAP1/β-catenin pathway (Fig. 10). Our studies suggest that KLF15/*LINC00689*/PTBP1/*LATS2* axis will be as a therapeutic potential target for CRC therapy.

Dysregulated KLF15 expression appears in many diseases^11,38^, and the anti-tumor effects of KLF15 have demonstrated in different type of cancers. For instance, breast cancer patients with high KLF15 expression were suggested to have a better outcome, and overexpression of KLF15 repressed the proliferation and migration of breast cancer cells, blocked cell cycle at G_0_/G_1_ phase^14^. Therefore, KLF15-based target therapy contributes to inhibit tumor growth, KLF15 is considered as an ideal tumor biomarker. Also, the downregulation of KLF15 in CRC was reported by Huang et al., but the detailed role and regulatory mechanism have not been studied^39^. Our results further confirmed this finding. But, in our results of Fig. 2a, it’s important to note that we intentionally seeded the cells at a relatively medium density initially, given the planned 72-h cultivation period. Upon closer examination of Fig. 2a, specifically in the KLF15 knockdown group, it became evident that the growth rate plateaued around the 72-h mark, and there was even a decline observed in LoVo cells. This observation suggested that cell density could indeed influence the growth rate. It is possible that the normal control group and the negative control group had not reached the same cell density threshold during the 72-h period, which may explain why their growth rates remained relatively consistent throughout the experiment. More importantly, we demonstrated that KLF15 acted as an upstream transcriptional regulator of *LINC00689* to increase its expression in CRC. The function of the KLF15/lncRNA axis has been discussed in other cancer pathogenesis. For example, KLF15 positively mediated lncRNA *TFAP2A-AS1* to suppress gastric cancer cell proliferation and migration^40^. Similarly, our findings suggested that the upregulated *LINC00689* mediated by KLF15 inhibited proliferation, migration, and invasion of CRC cells and suppressed the YAP1/β-catenin signaling pathway both in vitro and in vivo.

LncRNAs interact with RNA binding proteins to affect complex structure and regulatory outcomes in various tumor cells^41^. In this study, PTBP1, a known regulator of posttranscriptional gene expression that controls mRNA splicing, translation, stability, and localization^42^, was firstly found to act as a target protein of *LINC00689*, and *LINC00689* was strongly co-localized with PTBP1 in cytoplasm in HCT116 and LoVo cells. Several studies have also shown that cytoplasmic lncRNA-RNA binding protein interactions are involved in tumorigenesis. For instance, the functional ribonucleoprotein complex of UCA1 and PTBP1 promoted breast cancer growth by suppressing *p27* mRNA level^43^. LncRNAs may act as a scaffold for the formation of functional ribonucleoprotein complexes by multiple proteins, affecting the stability of mRNAs or accessibility to the translation machinery^44^. Here, one important finding is that overexpression of PTBP1 enhances the stability of *LATS2* mRNA, while knockdown of *LINC00689* reverses this effect, indicating that *LINC00689* stabilizes *LATS2* mRNA by recruiting PTBP1 protein.

Several publications have indicated that increased cell proliferative, metastatic progression, and tumorigenesis can result from abnormal Hippo activity^45,46^. LATS2 is one of the cores of the Hippo pathway, which restricts the activities of transcriptional co-activators, such as YAP1, followed by participating in a variety of cellular processes^47,48^. Hyperactivation of YAP1 is widespread in cancers. The ubiquitin-proteasome system breaks down the phosphorylated YAP1 in the cytoplasm, which prevents the production of YAP1 target genes required for cell migration and proliferation^49^. A previous publication has also revealed that YAP1 stimulated by E3 ubiquitin ligase SIAH2 interacts with HIF1α and is essential for HIF1α stability and function, thereby affecting cell proliferation and growth^50^. In our study, p-YAP1 (S127), and YAP1/β-catenin pathway target genes CTGF, CYR61, and HIF1α were also regulated by KLF15/*LINC00689*/PTBP1 axis. In addition, LATS2 is essential for the phosphorylation and deactivation of the transcription co-factor YAP1/TAZ in the Hippo signaling pathway^51^. In our previous study, *LINC00689* inhibited the activation of YAP1/β-catenin signaling pathway, thereby repressing the progression of CRC^7^. Herein, knockdown of LATS2 was demonstrated to reverse the inhibitory effect of *LINC00689* overexpression on YAP1/β-catenin pathway and CRC cell malignant phenotypes. Similar molecular mechanisms have been discussed in the publications of ovarian cancer^52^ and gastric cancer^53^.

One of the main constraints of this study is the primary focus, which mainly aims to elucidate the underlying molecular process and functional interaction within the cellular context. As such, our experiment design primarily revolves around in vitro models except for Fig. 4. We are well aware that performing more rescue experiments in animal models to validate the importance of KLF15/*LINC00689*/PTBP1/*LATS2* axis will provide a more holistic understanding and translational relevance of the observed molecular interaction. However, we were unable to conduct extensive in vivo investigations within the timeframe and resources allocated for this study. The more in vivo rescue experiments will be our future work. Another limitation is that we have not used EMSA assay to confirm *LINC00689* binding with PTBP1, adopting this method will accurately strengthen our conclusion.

Importantly, the therapeutic relevance of the KLF15/*LINC00689*/PTBP1/*LATS2* axis in the treatment of CRC is underscored by the regulatory effects of certain drugs on KLF15 expression. Several studies have shown that drugs such as Diallyl trisulfide, Allicin and Resveratrol exert modulatory effects on KLF15 expression^54–56^. Interestingly, these drugs have also been reported to inhibit the development of CRC^57–59^. We therefore speculated that KLF15/*LINC00689*/PTBP1/*LATS2* axis might serve as a crucial molecular pathway involved in the response to certain therapeutic agents in CRC. Meanwhile, commercially available drugs targeting the elements of this axis will become the candidate therapeutic drug to CRC treatment.

In conclusion, KLF15 and *LINC00689* were downregulated in CRC and showed a strong correlation, indicating the potential function to act as diagnostic and therapeutic indexes in CRC. Mechanistically, KLF15 bound to *LINC00689* promoter to upregulate its expression, which inhibited the activation of YAP1/β-catenin pathway through stabilizing *LATS2* mRNA by interacting with PTBP1 protein. These findings revealed the function of KLF15/*LINC00689*/PTBP1/*LATS2* axis in CRC and provided a viable theoretical basis for the development of better targeted therapies.

## Methods

### Clinical specimens

The protocol of the present study was approved by the Ethics Committee of Xiangya Third Hospital, Central South University, and informed consent was obtained from each patient. Total 28 paired CRC tissues and adjacent normal tissues were collected from patients who underwent surgical operation at the Xiangya Third Hospital, Central South University, and then frozen in −80 °C for subsequent experiments. All patients did not undergo chemoradiotherapy before and the clinical pathological traits of these patients were shown in Supplementary Table 1.

### Bioinformatic analyses

*KLF15* levels in CRC patients and the relationship between *KLF15* level and clinicopathological parameters (individual cancer stages and nodal metastasis status) of CRC patients were investigated through UALCAN (https://ualcan.path.uab.edu/index.html). The potential binding sites between KLF15 and *LINC00689* promoter were predicted by hTFtarget database (http://bioinfo.life.hust.edu.cn/hTFtarget#!/). The potential target proteins of *LINC00689* and potential binding between PTBP1 and *LATS2* mRNA were predicted by StarBase (https://starbase.sysu.edu.cn/index.php).

### Cell culture

CRC cell lines HCT116 and LoVo were obtained from the American Type Culture Collection (Manassas, VA, USA) and maintained in McCoy’s 5A medium supplemented with 10% fetal calf serum (Gibco, Grand Island, NY, USA) and streptomycin/penicillin (Millipore, Bedford, MA, USA) at 37°C in 5% CO_2_ environment.

CRISPR/Cas9 technology was utilized to knockout (KO) the *LINC00689* gene in HCT116 and LoVo cells. *LINC00689*-KO HCT116 and LoVo cells were purchased from Starfish Biotechnology (Suzhou, China). All the cell lines included in this study have been authenticated by STR profiling and tested for mycoplasma contamination.

### Cell transfection

The constructs used to knock down *LINC00689*, PTBP1, or LATS2 (sh-*LINC00689*, sh-PTBP1, or sh-LATS2) were synthesized and obtained from GenePharma (Shanghai, China). To obtain the overexpression vectors (OE-*LINC00689* and OE-PTBP1), *LINC00689* or PTBP1 was inserted into pcDNA3.1. All the plasmids were transfected into HCT116 or LoVo cells using Lipofectamine 3000 (Invitrogen, Carlsbad, CA, USA). A lentiviral system was used to silence or overexpress KLF15, and GenePharma provided a protocol for lentiviral production and infection. All sequences related to shRNAs are shown in Supplementary Table 2.

### Luciferase reporter assay

The *LINC00689* promoter sequence containing KLF15 seed matching sites (*LINC00689*-WT) was amplified via PCR and cloned into the pGL3-basic luciferase vector (GeneChem, Shanghai, China). The mutant reporter plasmid was also generated by mutating KLF15 seed site sequences (*LINC00689*-MUT). HCT116 and LoVo cells seeded in 6-well plates and grown to around 80% confluence, then cells were co-transfected with OE-KLF15 or OE-NC plasmids and recombinant reporter plasmids (*LINC00689*-WT/MUT) for 48 h using Lipofectamine 3000 (Invitrogen) according to the manufacturer’s protocol. Afterwards, cells were harvested and luciferase activities were measured using a Luciferase Reporter Assay Kit (Promega, Madison, WI, USA). Briefly, the transfected HCT116 and LoVo cells were lysed and centrifuged to remove cell debris. The supernatant was transferred to a luminometer plate and luciferase substrate from the Luciferase Reporter Assay Kit was added. Luciferase activity was measured using a luminometer (Thermo Fisher Scientific).

### Chromatin immunoprecipitation (CHIP)

CHIP Assay Kit (17-295, Millipore) was used to verify the interaction between KLF15 protein and *LINC00689* promoter. Briefly, cells were cross-linked with 1% formaldehyde for 10 min, then lysed and sonicated to obtain chromatin fragments. The chromatins diluted by CHIP solution were immunoprecipitated with KLF15 (1:200, ab2647, Abcam, Cambridge, UK) or IgG (1:100, ab172730, Abcam) antibody at 4 °C overnight with rotation. After reversing the cross-links, the complexes were purified and analyzed by qPCR using SYBR Green Supermixes (Bio-Rad). The primers of *LINC00689* promoter were described in Supplementary Table 3.

### Electrophoretic mobility shift assay (EMSA)

The biotin-labeled probe of *LINC00689* promoter region was made by PCR and then purified to remove any unincorporated labels or contaminants. The *KLF15* coding sequence was cloned into a bacterial expression vector and transformed into E. coli cells. The bacteria were induced to express the recombinant KLF15 protein. The recombinant KLF15 protein was purified from the bacterial lysates using nickel affinity chromatography, eluted with imidazole, and dialyzed into a suitable buffer for storing the purified protein. Afterwards, the EMSA was performed using a EMSA Kit (E33075, Invitrogen). In brief, the biotin-labeled DNA probe was incubated with purified recombinant KLF15 protein in binding buffer from the EMSA kit. The protein-DNA complexes were separated by electrophoresis on 6% polyacrylamide gel. The biotin-labeled DNA was then transferred to a nylon membrane and detected using streptavidin-horseradish peroxidase and chemiluminescent substrate provided in the kit.

### Measurement of stability of *LATS2* mRNA

To determine the effect of *LINC00689* and PTBP1 expression on the decay rate of *LATS2* mRNA, HCT116 and LoVo cells silenced *LINC00689* and overexpressed PTBP1 were incubated with Actinomycin-D (Act-D, 2 μg/mL, Sigma-Aldrich, St. Louis, MO, US). Cells were harvested and RNA was isolated at indicated time (t = 4, 8, 12, 24 h) after adding Act-D. The stability of *LATS2* mRNA was analyzed via RT-qPCR.

### Cell counting kit-8 (CCK-8) assay

HCT116 and LoVo cells transfected with OE-KLF15, sh-KLF15, OE-KLF15 + sh-*LINC00689*, OE-*LINC00689*, and OE-*LINC00689* + sh-LATS2 (2×10^4^ cells/mL) were cultured in 96-well plates overnight. After adding CCK-8 solution (Sigma-Aldrich) and incubating for another 2 h, cell proliferation was assessed every 24 h using microplate reader at a wavelength of 450 nm.

### Wound healing assay

Treated cells were seeded in 24-well plates, and an artificial wound was created using a 200-μL pipette tube. Cells were then cultured in serum-free medium for 24 h, and then the wound closure was observed and imaged under a microscope (Zeiss, Germany). We measured the fraction of cell coverage across the line for evaluate the migration rate.

### Transwell assay

Cell invasion and migration abilities were analyzed using Transwell chambers (8 µm, Corning Incorporated, USA) with or without Matrigel (BD Bioscience, San Jose, CA, USA) coating. Briefly, cells transfected with indicated oligonucleotides were resuspended in serum-free medium and added to the upper chamber. The lower chamber contained complete medium supplemented with fetal calf serum as a chemoattractant. After 48 h of incubation at 37°C, cells that had invaded or migrated through the membranes were fixed with 4% paraformaldehyde and stained with crystal violet. At last, the invaded or migrated cells were imaged under a microscope and the number of stained cells was counted.

### Immunofluorescence

Treated cells were fixed in 4% paraformaldehyde (Santa Cruz Biotechnology, Dallas, Texas, USA) for 20 min at room temperature and then washed twice in PBS to remove residual paraformaldehyde. Afterwards, cells were permeabilized with 0.1% Triton X-100 made in PBS solution for 15 min, followed by blocking with 2% BSA for 1 h. Cells were washed with PBS prior to being incubated with anti-N-cadherin primary antibody (1:1000, ab18203, Abcam) overnight at 4 °C and cultured with secondary antibody (1:3000, ab6717, Abcam) for 1 h at room temperature. Cellular nuclei were counterstained with DAPI (Santa Cruz Biotechnology). Cells were detected with a fluorescence microscope (Zeiss).

### RNA immunoprecipitation (RIP)

RIP was performed to confirm the potential RNA-binding proteins of *LINC00689* by a Magna RIP RNA-Binding Protein Immunoprecipitation Kit (Millipore) according to the manufacturer’s protocol. Briefly, cell lysis was conducted using RIP lysis buffer containing RNase and proteinase inhibitor, and then incubated with anti-U2AF2 (1:50, #70471, Cell Signaling Technology, Beverly, MA, USA), anti-SRSF1 (1:150, #32-4500, Thermo Fisher Scientific, Waltham, MA, USA), anti-PTBP1 (1:50, #32-4800, Thermo Fisher Scientific), anti-UPF1 (1:50, #9435, Cell Signaling Technology), or anti-IGF2BP2 (1:100, ab128175, Abcam) controlled by normal rabbit IgG (1:100, ab172730, Abcam) at 4°C overnight. After treatment with proteinase K buffer, the immunoprecipitated RNAs were extracted using the RNeasy MinElute Cleanup Kit (Qiagen, Duesseldorf, Germany), and then the enrichment of *LINC00689* was measured by RT-qPCR.

### RNA pull-down

Biotin-labeled *LINC00689* was transcribed with Biotin RNALabeling Mix and T7 RNA polymerase, treated with RNase-free DNase I (Roche) and purified with a RNeasy Mini Kit. Total RNA was heated and annealed to form secondary structure, mixed with cytoplasm extract in RIP buffer at room temperature for 1 h. Afterwards, the biotinylated lncRNAs were captured with streptavidin magnetic beads. The mixture was washed and eluted. The eluate was subjected to western blotting analysis.

### Fluorescence in situ hybridization (FISH)

FISH assay was conducted to analyze the co-localization of *LINC00689* and PTBP1 in HCT116 and LoVo cells. Briefly, HCT116 and LoVo cells with *LINC00689* knockout or not grown on the slides were washed with PBS and fixed in 4% paraformaldehyde. Labeled *LINC00689* probes were designed and synthesized by RiboBio (Guangzhou, China). Probes were mixed with pre-made hybridization buffer, and then samples were incubated in hybridization buffer at 37 °C overnight. After washed with hybridization buffer at 37 °C for 15 min, cells were stained with DAPI. Images were obtained under fluorescence microscope (Zeiss).

### Tumorigenesis in nude mice

The nude BALB/C mice (4–6 weeks, male, 18–22 g) were purchased from Hunan SJA laboratory animal CO., LTD (Changsha, Hunan, China) and randomly divided into four groups (n = 8): sh-NC, sh-KLF15, OE-NC, and OE-KLF15. According to the grouping needs, mice were subcutaneously injected with HCT116 or LoVo cells (5 × 10^5^ cells in 0.2 mL PBS/mouse) infected with sh-KLF15 or OE-KLF15 lentiviral vector. After 25 days, the mice were sacrificed, and tumors were excised. Tumor volumes were acquired by measuring tumor length and width using a slide gauge. Mice were maintained in specific pathogen-free conditions and all research work was approved by the Institutional Animal Care and Use Committee of Xiangya Third Hospital, Central South University.

### Immunohistochemistry

The isolated tumor tissues were fixed in 10% buffered formalin, dehydrated and embedded in paraffin. The immunohistochemistry reaction was performed on 4-μm-thick paraffin sections fixed to microscopic slides. Briefly, sections were deparaffinized with xylene twice then subjected to antigen retrieval in citrate buffer (pH 6.0) at 98 °C for 15 min. Subsequently, sections were incubated with primary antibodies: anti-Ki-67 (1:1000, ab15580, Abcam), anti-E-cadherin (1:1000, ab231303, Abcam), or anti-N-cadherin (1:2000, ab76011, Abcam) at 4°C overnight. After incubated with biotinylated secondary antibodies (1:3000, ab205719 and ab6721, Abcam), the reaction products were stained with 3,3’-diaminobenzidine and counterstained with haematoxylin at room temperature for 2 min. A light microscope (Zeiss) was used to observe the slides under magnification (200×).

### Reverse transcription-quantitative PCR (RT-qPCR)

Total RNAs of treated tissues or cells were extracted with Trizol reagent (Invitrogen) following the manufacturer’s instruction, and then was reverse-transcribed to cDNA with PrimeScript RT Master Mix (Takara, Tokyo, Japan). RT-qPCR detection of *KLF15*, *LINC00689*, and *LATS2* mRNA expression levels was performed with SYBR Green Master Mix (Applied Biosystems, Carlsbad, CA, USA) according to the manufacturer’s instruction on ABI7900HT (Thermo Fisher Scientific). GAPDH was regarded as the internal control. The gene expression levels were presented as fold changes relative to the expression level of internal control using the 2^–ΔΔCt^ method: 2^−ΔΔCt^ = 2^−[ΔCt (sample)−ΔCt (control)]^ (Eq. (1)). The primers used in this method are described in Supplementary Table 3.

### Western blotting

Proteins were extracted from cells and tissues, and the concentration was quantified by the BCA protein assay Kit (Thermo Fisher Scientific). Equal amount of proteins from each group was purified with 10% SDS-polyacrylamide gel electrophoresis, then transferred to a polyvinylidene difluoride (PVDF) membrane. Blocking of non-specific binding was achieved by incubation over 1 h with 5% skim milk. Next, the membrane was incubated with primary antibodies overnight at 4 °C, followed by treatment for 1 h with horseradish peroxidase-conjugated secondary antibody (1:1000, #7074 and 1:2000, #7076, Cell Signaling Technology). The antibody-reactive bands were detected with ECL reagent (Millipore). GAPDH (1:2000, ab8245, Abcam) and β-tubulin (1:2000, ab6046, Abcam) were used as internal controls. For the detection of close protein bands in the same membrane, “membrane stripping” method was performed to remove the previous primary and secondary antibodies via washing with Stripping buffer (Thermo Fisher Scientific), then the blot was reprobed. The following primary antibodies were used: anti-KLF15 (1:2000, ab2647, Abcam), anti-PTBP1 (1:5000, ab133734, Abcam), anti-LATS2 (1:1000, #5888, Cell Signaling Technology), anti-CTGF (1:1000, ab209780, Abcam), anti-CYR61 (1:2000, ab228592, Abcam), anti-HIF1α (1:1000, ab179483, Abcam), anti-p-YAP1 (S127, 1:5000, ab76252, Abcam), anti-YAP1 (1:1000, ab56701, Abcam), anti-β-catenin (1:5000, ab32572, Abcam), anti-Slug (1:1500, ab27568, Abcam), and anti-Vimentin (1:1000, ab92547, Abcam).

### Statistics and reproducibility

All experiments were performed in at least three biological replicates, and each biological replicate contained three technical replicates. GraphPad Prism 8.0 (GraphPad Software, San Diego, CA, USA) was used for statistical analyses. The data were presented as mean ± standard deviation (SD). All the data meet the assumption of normal distribution. Student’s t-test was performed to evaluate the differences between two groups, one-way analysis of variance (ANOVA) with Tukey post hoc test was performed to determine significant differences between multiple groups. The results were considered statistically significant at *P* ≤ 0.05.

### Supplementary information

Supplementary Information

Description of Additional Supplementary Files

Supplementary Data 1

## Data Availability

The sequences of shRNAs, primers used in CHIP and RT-qPCR methods, all of the uncropped images in western blotting were shown in Supplementary Fig. 1. The source data behind the graphs in the manuscript were shown in Supplementary Data 1. The other data generated during and/or analyzed during the current study are available from the corresponding author upon reasonable request.
