# Supplementary material for: KLF15 transcriptionally activates LINC00689 to inhibit colorectal cancer development
Source: Commun Biol. 2024 Jan 25;7:130. doi: 10.1038/s42003-023-05757-3 (PMC10810960; doi:10.1038/s42003-023-05757-3)
Supplement: Supplementary file 1 — Supplementary Information [file 42003_2023_5757_MOESM1_ESM.pdf]

Supplementary table 1 Correlation between KLF15 expression and clinicopathlogical characteristics of colorectal cancer

Clinical parameter	KLF15 expression		*P*- Value
	Low (n=14)	High (n=14)	
Age (years)			
< 50	7	9	0.7036
≥ 50	7	5	
Gender			
Male	6	8	0.7064
Female	8	6	
Tumor size (cm)			
< 5	10	5	0.1283
≥ 5	4	9	
Location			
Distal	6	5	0.7040
Middle	3	5	
Proximal	5	4	
Histologic differentiation			
Well or moderate	6	10	0.1266
Poor	8	4	
TNM stage			
I-II	4	11	0.0080
III-IV	10	3	

Supplementary table 2 Targets and sequences related to shRNAs used in cell transfection

LINC00689-SH	GGATGAGAATACACGCCTTCC
S	AATTGGGATGAGAATACACGCCTTCCTTCAAGAGAggaaggcgtgtattctcatccTTTTTT
A	GATCAAAAAAGGATGAGAATACACGCCTTCCTCTCTTGAAggaaggcgtgtattctcatccC

PTBP1-SH1	GATATAGCCGTTGGTACAAAG
S	AATTGGATATAGCCGTTGGTACAAAGTTCAAGAGActttgtaccaacggctatatcTTTTTT
A	GATCAAAAAAGATATAGCCGTTGGTACAAAGTCTCTTGAActttgtaccaacggctatatcC

PTBP1-SH2	GCACAGTGTTGAAGATCATCA
S	AATTGGCACAGTGTTGAAGATCATCATTCAAGAGAtgatgatcttcaacactgtgcTTTTTT
A	GATCAAAAAAGCACAGTGTTGAAGATCATCATCTCTTGAAtgatgatcttcaacactgtgcC

LATS2-SH	GCAGAAGTGAACCGGCAAATG
S	AATTGGCAGAAGTGAACCGGCAAATGTTCAAGAGAcatttgccggttcacttctgcTTTTTT
A	GATCAAAAAAGCAGAAGTGAACCGGCAAATGTCTCTTGAAcatttgccggttcacttctgcC

Supplementary table 3 The primers used in CHIP and RT-qPCR methods

h-LINC00689-135-F (CHIP)	CGGGCGCAGAGACAGAGGA
h-LINC00689-135-R (CHIP)	GCTGCGTCCTCTGTCTCTG
h-LINC00689-219-F	CATGCCCTCCCTTCCATCGC
h-LINC00689-219-F	GGTCTGCAGCGATTGAAAGGAG
h-PTBP1-204-F	CTCTCCGTATGCAGGAGCTG
h-PTBP1-204-R	TCTGGGTTGAGGTTGCTGAC
h-LATS2-158-F	AAGAGCTACTCGCCATACGC
h-LATS2-158-R	ATCTTCCGCATCTGCTCCTG

Supplementary figure 1 Uncropped and unedited blot/gel images

1f

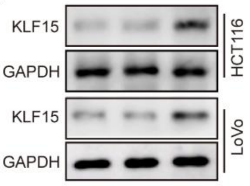

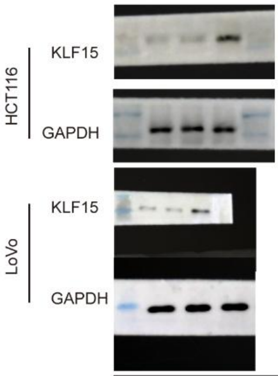

1i

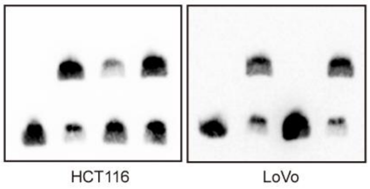

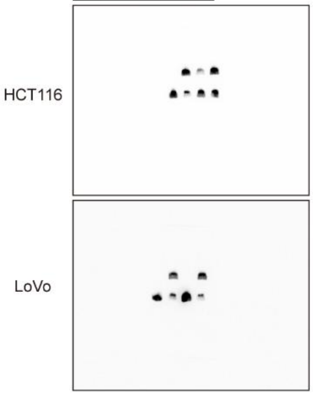

3b

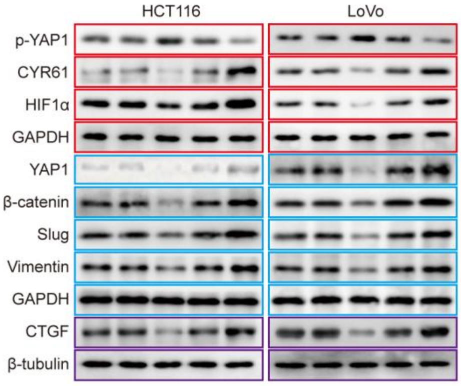

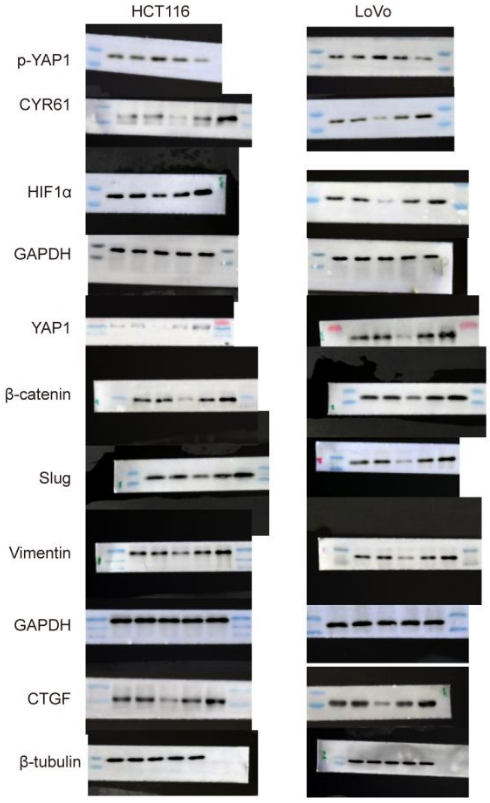

4g

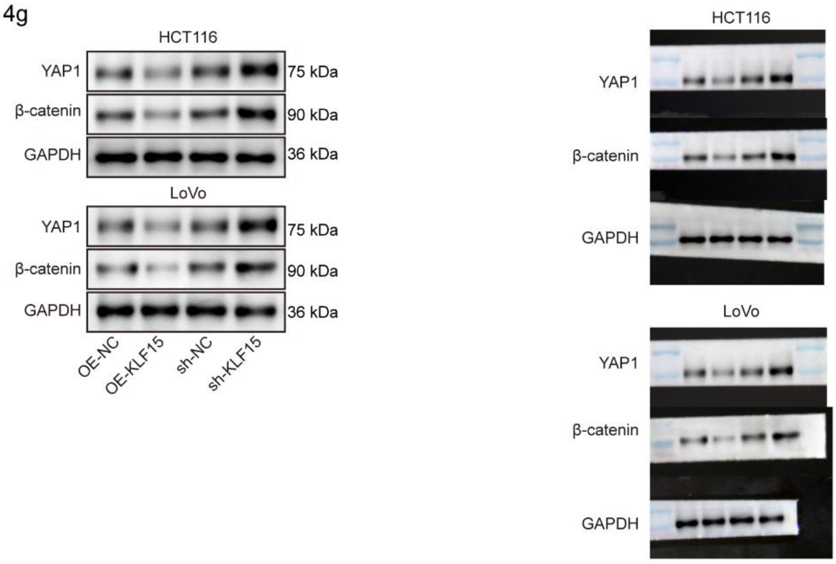
HCT116
YAP1
75 kDa
β-catenin
90 kDa
GAPDH
36 kDa
LoVo
YAP1
75 kDa
β-catenin
90 kDa
GAPDH
36 kDa
OE-NC
OE-KLF15
sh-NC
sh-KLF15
HCT116
YAP1
β-catenin
GAPDH
LoVo
YAP1
β-catenin
GAPDH

5e

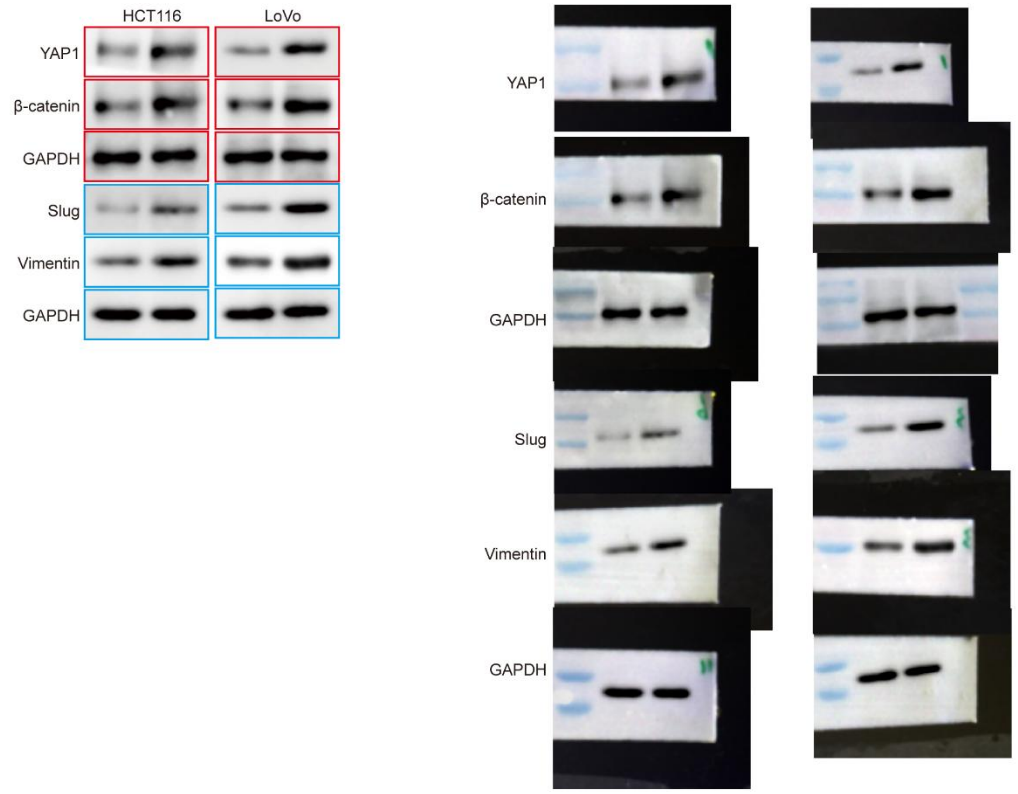
HCT116
LoVo
YAP1
β-catenin
GAPDH
Slug
Vimentin
GAPDH
HCT116
LoVo
YAP1
β-catenin
GAPDH
Slug
Vimentin
GAPDH

6g

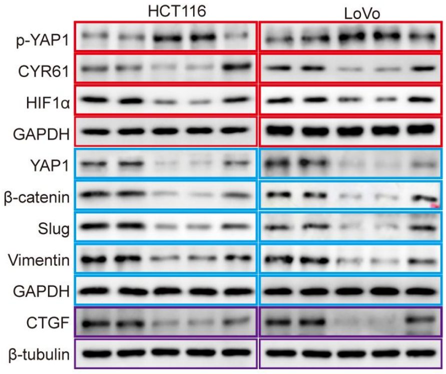
HCT116
LoVo
p-YAP1
CYR61
HIF1α
GAPDH
YAP1
β-catenin
Slug
Vimentin
GAPDH
CTGF
β-tubulin

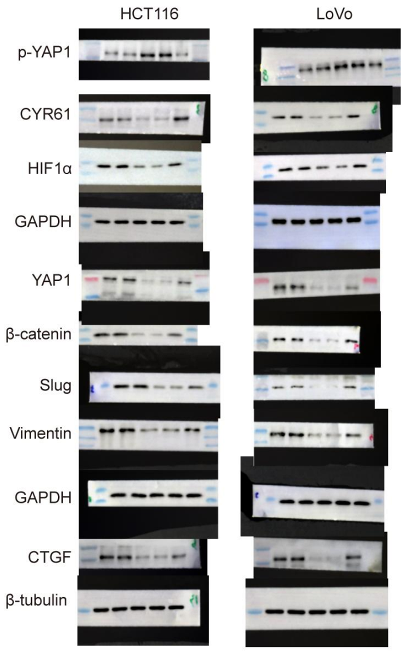
HCT116
LoVo
p-YAP1
CYR61
HIF1α
GAPDH
YAP1
β-catenin
Slug
Vimentin
GAPDH
CTGF
β-tubulin

7b

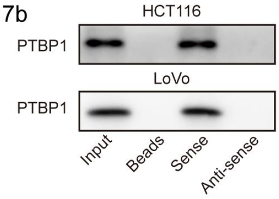
HCT116
PTBP1
LoVo
PTBP1
Input
Beads
Sense
Anti-sense

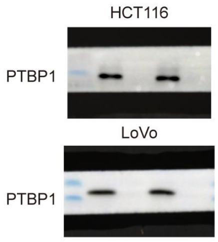
HCT116
PTBP1
LoVo
PTBP1

8c

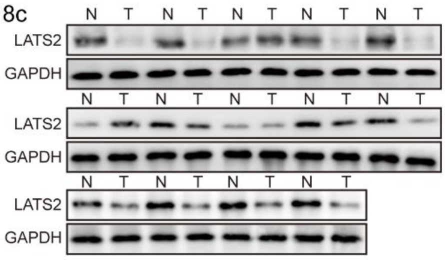
N T N T N T N T N T
LATS2
GAPDH
N T N T N T N T N T
LATS2
GAPDH
N T N T N T N T
LATS2
GAPDH

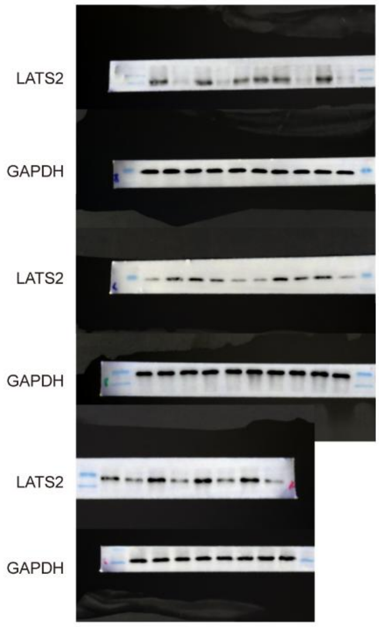
LATS2
GAPDH
LATS2
GAPDH
LATS2
GAPDH

8g

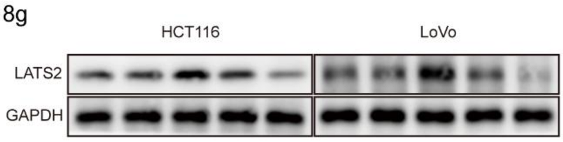
HCT116
LoVo
LATS2
GAPDH

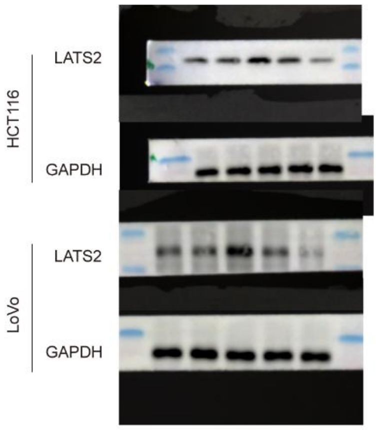
HCT116
LATS2
GAPDH
LoVo
LATS2
GAPDH

9b

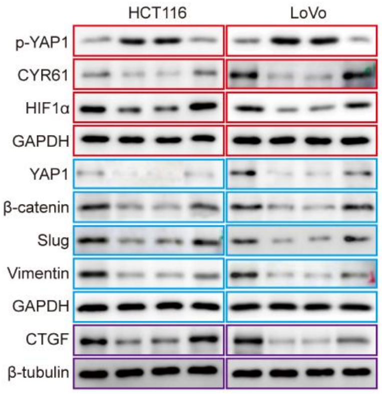
HCT116
LoVo
p-YAP1
CYR61
HIF1α
GAPDH
YAP1
β-catenin
Slug
Vimentin
GAPDH
CTGF
β-tubulin

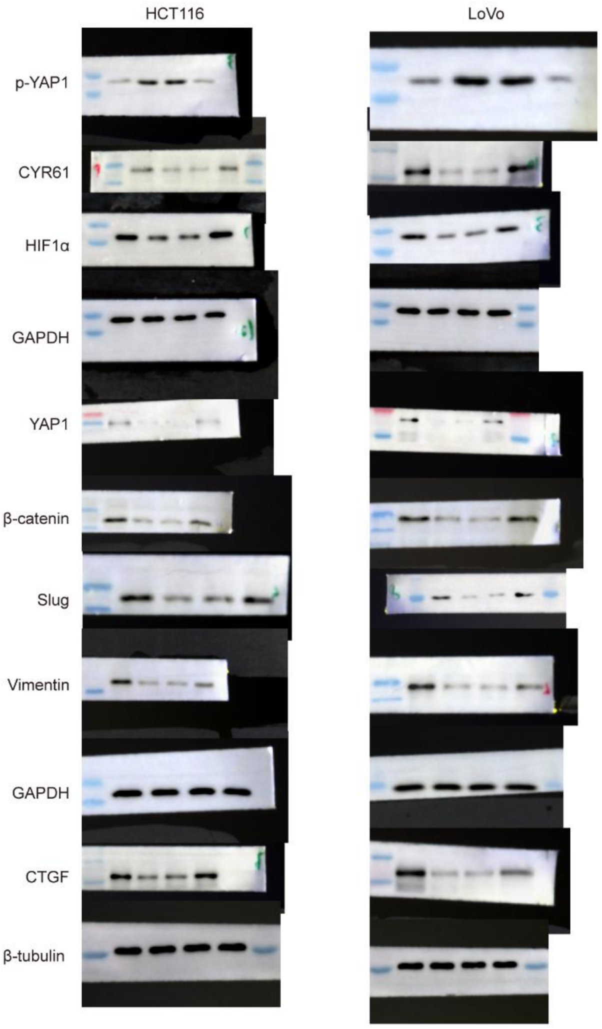
HCT116
LoVo
p-YAP1
CYR61
HIF1α
GAPDH
YAP1
β-catenin
Slug
Vimentin
GAPDH
CTGF
β-tubulin
