# Supplementary material for: KLF15 transcriptionally activates LINC00689 to inhibit colorectal cancer development
Source: Commun Biol. 2024 Jan 25;7:130. doi: 10.1038/s42003-023-05757-3 (PMC10810960; doi:10.1038/s42003-023-05757-3)
Supplement: Supplementary file 2 — Description of Additional Supplementary Files [file 42003_2023_5757_MOESM2_ESM.pdf]

Description of Additional Supplementary Files

File Name: Supplementary Data 1
Description: Source data underlying figures.
